# 18F-FDG PET/CT- and MRI-Based Locally Advanced Cervical Cancer Early-Response Assessment after Concurrent Chemo- and Radiotherapy—Impact on Patient Outcomes and Survival Prediction

**DOI:** 10.3390/diagnostics14131432

**Published:** 2024-07-04

**Authors:** Silvija Lucic, Milena Spirovski, Dragana Stojanovic, Andrea Peter, Jelena Licina, Olivera Ivanov, Natasa Milenovic, Milos A. Lucic

**Affiliations:** 1 Medical Faculty, University of Novi Sad, 21000 Novi Sad, Serbia; milena.spirovski@mf.uns.ac.rs (M.S.); andrea.peter@mf.uns.ac.rs (A.P.); jelena.licina@mf.uns.ac.rs (J.L.); olivera.ivanov@mf.uns.ac.rs (O.I.); natasa.milenovic@mf.uns.ac.rs (N.M.); milos.lucic@mf.uns.ac.rs (M.A.L.); 2 Oncology Institute of Vojvodina, 21000 Novi Sad, Serbia; draganadasa94@yahoo.com

**Keywords:** cervical cancer, 18F-FDG PET/CT, MRI, post-radiotherapy response, survival

## Abstract

With one third of patients with locally advanced cervical cancer (LACC) expected to develop cancer recurrence in the first two years after therapy, accurate assessment of the response and timely detection of cancer recurrence after concurrent chemo- and radiotherapy (CCRT) treatment is of great importance. Although there is neither definite consensus about the preferred imaging modality, nor the time interval until the first diagnostic examination after CCRT, the National Comprehensive Cancer Network (NCCN) recommends the use of MRI and 18F-FDG PET/CT as a post-treatment LACC response-assessment imaging tools. In this study, we tried to appraise the early therapy response in LACC patients by both 18F-FDG PET/CT and MRI in regard to the follow-up imaging results and their mutual interrelationship, and to ascertain if the post-treatment 18F-FDG PET/CT and MRI results were related to the progression-free and overall survival rate in women with LACC after CCRT. We also aimed to estimate the early and follow-up diagnostic imaging impact on further therapy management. Based on our results, we concluded that 18F-FDG PET/CT did surpass MRI in the early assessment of therapeutic response in LACC patients after CCRT. Both modalities provided information that may serve as predictive biomarkers of outcome and LACC patients’ survival.

## 1. Introduction

Cervical carcinoma is the fourth-most-frequent cancer, and the fourth leading cause of cancer deaths in women worldwide. It is also the third leading cause of cancer deaths among women in less-developed countries. Despite being one of the most preventable neoplasms, and already the second-most-frequently diagnosed malignancy in women, cervical cancer remains the second leading cause of cancer death in women of younger age (20–39 years) with considerable disparities that characterize the incidence and mortality with increased rates in low- and middle-income countries [1,2,3].

The World Health Organization recognizes three pathohistological categories of epithelial cervical tumors: squamous, glandular (adenocarcinoma), and other epithelial tumors (adenosqamous, neuroendocrine tumors, and undifferentiated carcinoma), where squamous cell carcinomas account for 70–80%, and adenocarcinomas for 20–25% of all tumors [4,5].

Early cervical cancer and locally advanced cervical cancer (LACC) have distinct therapeutic approaches and prognoses, where concurrent chemoradiation is the standard treatment option for patients with locally advanced bulky IB2–IVA disease, in recent FIGO updates declared as stage IB3–IVA, but also recommended for stages IB1–2, if other treatment options are unacceptable [6,7,8].

The external beam radiotherapy (EBRT) field includes the uterus, parametrial tissue, part of the vagina, and pelvic lymph nodes, and can be extended to the level of the paraaortic region if needed, followed by a brachytherapy boost as a preferred option for the possible residual cancer [9].

Treatment response surveillance for residual or recurrent disease after chemoradiation therapy is of utmost importance, with understanding of this feature being an uppermost prognostic factor for disease progression-free survival (PFS) and overall survival (OS) [10,11,12].

Since one third of patients are expected to develop cancer recurrence during the first two years after therapy, it is of paramount importance to detect it as early as possible, and to conduct salvage therapy in order to improve the outcome [9,13,14].

Cancer-recurrence appearance is more frequently locoregional, rather than the much less common, distant metastatic presentation. Recommendations for response evaluation after treatment include using clinical and imaging techniques to provide evaluation at 3–6 months.

Even though there is evidence that a three-month posttherapy restaging with 18F-FDG PET/CT correlates highly with prognosis, there is no definitive agreement on the modality of choice for early therapy-response evaluation [15].

ESGO/ESTRO/ESMO guidelines recommend that the same imaging modality used at the start of treatment should be used for tumor-response evaluation, and that imaging should not be performed earlier than 3 months after the end of treatment; however, in cases of uncertain complete remission, assessment should be repeated after 2–3 months [15,16].

Without a clear consensus on the gold-standard modality, MRI is usually considered and used as the modality of choice for the first imaging technique in therapy-response evaluation, and 18F-FDG PET/CT is mostly performed if residual/recurrent disease is suspected by MRI [14,17,18,19,20].

In the absence of a definite agreement on the preferred imaging technique, partly due to the lack of fully reliable and accurate data, the National Comprehensive Cancer Network (NCCN) recommends the use of MRI and PET/CT for the post-treatment assessment of tumor response in locally advanced cervical cancer (LACC) patients after concurrent chemo- and radiotherapy (CCRT) [9,21].

Since a number of prospective trials have evaluated 18F-FDG PET/CT in posttherapy response assessment, and have indicated it to be predictive of patients’ survival [17,22,23], in this study we tried to assess the early therapy response by MRI and 18F-FDG PET/CT in regard to the follow-up diagnostic imaging results and their mutual interrelationship, and to ascertain if the posttreatment 18F-FDG PET/CT results were related to the progression-free survival (PFS) and overall survival (OS) rate in women with LACC after CCRT. We also aimed to ascertain the early and follow-up diagnostic imaging impact on further therapy management.

## 2. Materials and Methods

### 2.1. Patients

Forty-one female Caucasian patients (mean age 53.39 ± 11.85 years; range 31–77; median 56) with biopsy-proven cervical cancer and radiologically determined International Federation of Gynecology and Obstetrics (FIGO) IB2–IVA disease stage were included in the study in the period from December 2020 to June 2022.

Study inclusion criteria were: (a) biopsy-proven cervical carcinoma, (b) cancer stage IB3 (+IB2–IVA, if other treatment options were unacceptable) according to the FIGO classification determined by pelvic and abdominal MRI examinations, performed after biopsy and before treatment initiation, (c) pretherapy MRI confirmation that the cancer was confined to the pelvic cavity, and (d) no prior chemotherapy or radiotherapy treatment.

The study was approved by our institutional ethical committee, and written informed consent was obtained from all patients.

### 2.2. Treatment

All forty-one patients underwent concurrent chemoradiation (CCRT) with curative intention and consisting of combined conformal external beam radiotherapy (EBRT) with concurrent chemotherapy and concomitant high-dose-rate (HDR) intracavitary brachytherapy (ICBT).

The standard EBRT dose was 45 Gy in 25 fractions, and HDR ICBT was delivered with an overall dose of 6000 cGy in 5 fractions (in accordance with the International Commission on Radiation Units and Measurements Reports 62 and 38). All of the patients were simultaneously treated with four-cycle cisplatin-based chemotherapy (40 mg/m^2^).

Early therapy response was assessed by pelvic and abdominal MRI in the period from 2 to 3 months, and 18F-FDG PET/CT in the period from 2 to 4 months after completion of combined irradiation. Follow-up MRI studies of the pelvis and abdomen were performed at 6 months and 12 months after therapy completion.

Patients with either local or distant metastatic progression after early imaging, confirmed by both modalities, underwent immediate re-irradiation and/or chemotherapy, regardless to the further control-study time schedule.

Patients with progressive disease detected in later imaging studies were further treated with chemotherapy.

### 2.3. Imaging Techniques

MRI examinations were performed on a 1.5 MR scanner (Aera, Siemens Health Care, Erlangen, Germany) with a body and pelvic phased-array coil using standardized institutional protocols, that included the following sequences: T1w coronal and T2w coronal tomograms with fat saturation (FS); T1w and T2w axial tomograms; diffusion-weighted image (DWI) axial tomograms with calculated apparent diffusion coefficient (ADC) map; T2w sagittal tomograms; targeted thin T2w tomograms perpendicular to the long axis of the cervix; and targeted T1w parasagittal tomograms on the para-iliac regions (for assessment of the lymph nodes).

18F-FDG PET/CT scans were acquired on PET/CT scanners (Siemens Biograph 64, Erlangen, Germany or GE Discovery MI DR, Chicago, IL, USA), combined with a low-dose CT from the base of the skull to the upper thigh level 60–90 min after the injection of 3.7 MBq/kg of 18F-FDG. Patients were required to fast for at least 4–6 h before examination and to have a blood glucose level below 7 mmol/L (with an exception for diabetic patients, where a glucose level below 11 mmol/L was required).

### 2.4. Statistical Analysis

Statistical analyses were carried out by use of SPSS statistics for Windows (version 28.0, IBM Inc., New York, NY, USA). Continuous variables are presented as means and medians with ranges, and categorical variables as frequencies with percentages. Descriptive statistics were used to summarize demographic data, treatment evaluation, and outcome. Two-sample z-test and Cohen’s kappa test were used to compare categorical variables.

Kaplan–Meier survival curves and log-rank test were used to analyse PFS and OS, and hazard ratios were calculated using Cox regression analysis, adjusted for age at diagnosis; FIGO stage; histology; tumor grade; and MRI and 18F-FDG PET/CT results. Modalities’ diagnostic-test characteristics were calculated on a patient-based level. Statistical tests were two-sided, and a *p* value of <0.05 was considered as statistically significant.

## 3. Results

Patients’ demographic data, staging with FIGO classification, histopathological cancer characteristics, recurrence status, and follow-up data are displayed in Table 1.

Generally, a whole group could be dichotomized into patients with low FIGO I–II stage (58.5%) and high FIGO III–IV stage (41.5%).

Diagnostic results at three observed checkpoints, starting with early abdominal and pelvic MRI and early 18F-FDG PET/CT, followed by control MRI examinations at 6 months and 12 months, are presented in Table 2.

Diagnostic accuracy was counted on a patient basis and calculated as presented in Table 3.

A two-sample z-test was used to test the difference between all obtained results. Cross-examined diagnostic features did not demonstrate a statistically significant difference.

Cohen’s Kappa coefficient between 18F-FDG PET/CT and 6-month follow-up was calculated as 0.62 (95% CI [0.434–0.805]), and between MRI and 6-month follow-up as 0.46 (95% CI [0.269–0.662]). Mutual agreement of MRI and 18F-FDG PET/CT was calculated as 0.698 (95% CI [0.514–0.882]).

By dividing patients into responder (CR) and non-responder (PR and PD) subgroups, we found that both imaging modalities demonstrated full agreement in defining complete response in the CR subgroup at all imaging checkpoints in 13 patients (31.71%), and full agreement in the PR/PD subgroup was found in 15 patients (36.58%). Discordant results were found in 13 patients. These are discussed in detail in Table 4.

**Figure 1:**
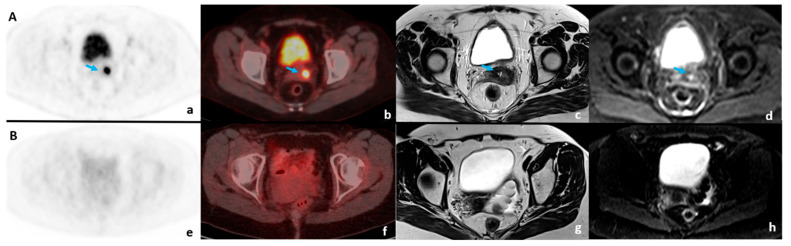
Concordant results of early CCRT posttreatment 18F-FDG PET/CT and MRI examinations are presented in row (**A**) (PR) and row (**B**) (CR). In row (**A**), residual cancer presence within a partial response is marked with a blue arrow on PET (**a**), fused PET/CT (**b**), axial T2w (**c**), and DWI (b = 800 s/mm^2^) (**d**) images. In row (**B**), complete therapy response is noticeable on PET (**e**), fused PET/CT (**f**), T2w (**g**), and DWI (b = 800 s/mm^2^) (**h**) images in the axial plane.

**Figure 2:**
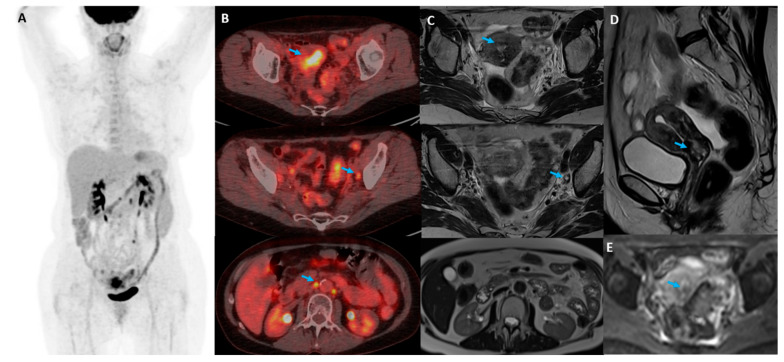
Posttreatment discordant early 18FDG PET/CT and MRI results with 6-month follow-up. Maximal intensity projection (MIP) 18F-FDG PET/CT reconstruction (**A**) indicates the presence of persistent metabolically active cervical cancer and pelvic and paraaortic lymph nodes, visible on fused PET/CT axial slices (column (**B**)) on three different levels (blue arrows). Residual cancer presence is indicated (blue arrows) on the same levels on T2w axial pelvic images (column (**C**)), T2w image in sagittal plane (**D**), and DWI (b = 800 s/mm^2^) image in axial plane (**E**). MRI failed to detect the presence of paraaortic lymph node metastasis.

Examples of concordant and discordant 18F-FDG PET/CT and MRI results are presented in Figure 1 and Figure 2, respectively.

In addition, 18F-FDG PET/CT detected 10 patients (24.39%) with distant metastasis; in six of them (14.63%), within the lung; in two (4.88%), in the liver; in one (2.44%), in the mediastinal lymph nodes; and in another one (2.44%), in supraclavicular lymph nodes.

During the total follow-up time, 23 patients (56.11%) died, out of these, 22 due to disease progression and one due to a treatment complication (pelvic abscess).

The OS rate for the whole group was 43.9%, with an OS rate of 88.2% for the CR group and 12.5% for the PD group.

The overall PFS rate for the whole group was 36.6%, with a PFS rate of 88.2% for the CR group and 0% for the PD group.

PFS and OS rates at all checkpoints are presented in Table 5.

Statistical difference was found between the PFS rate for the early MRI CR group and the PFS rate for the 12-months control CR group (z-score 2.19; *p* = 0.028).

The mean calculated progression-free survival (PFS) for the whole group of patients was 22.45 + 1.94 (95% CI [18.64–26.25]), median of 18 months (95% CI [12.19–23.81]).

The mean overall survival (OS) for the whole group of patients was 28.34 + 1.39 months (95% CI [26.22–31.65]), with a median of 30 months (95% CI [24.56–35.43]).

Statistical difference was observed by Kaplan–Meier analysis between responders and non-responders, both for PFS and OS (*p* < 0.001). For responders, mean PFS time was 36.031 months (95% CI [33.114–38.948]), and for non-responders, 13.5 months (95% CI [11.35–15.65]). For responders, mean OS time was 37.111 months (95% CI [36.069–38.153]), and for non-responders, 23.88 months (95% CI [20.979–26.781]). Both 18F-FDG PET/CT and MRI results demonstrated a statistically significant impact on Kaplan–Meiercalculated PFS and OS survival time (*p* < 0.001).

**Figure 3:**
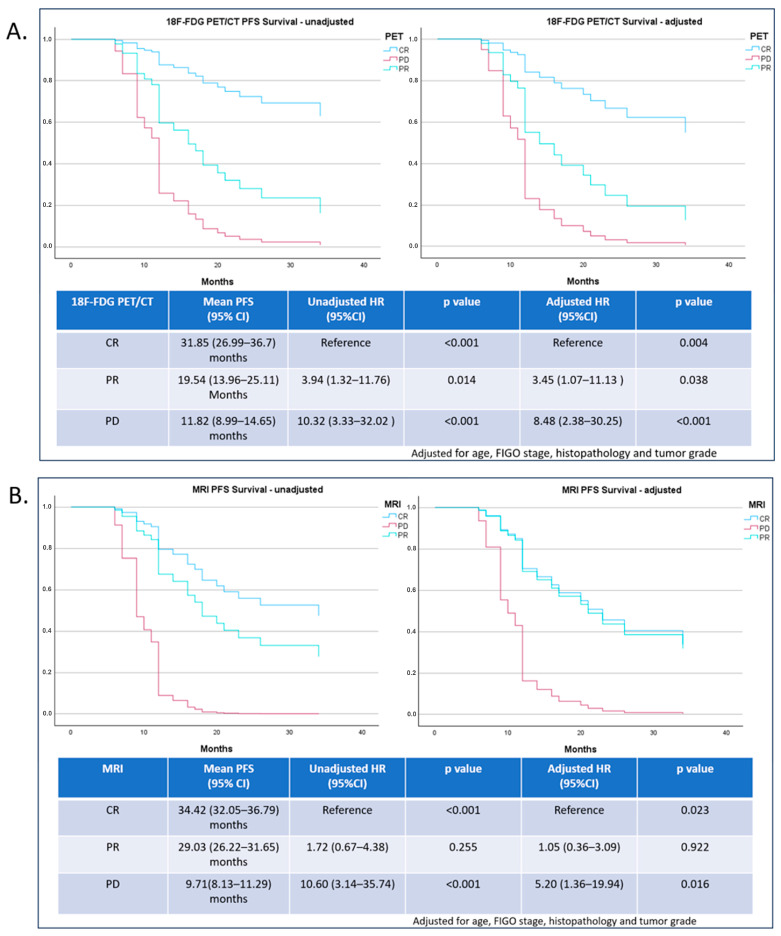
Progression-free survival as a function of 18F-FDG PET/CT (**A**) and MRI early therapy-response evaluation (**B**).

**Figure 4:**
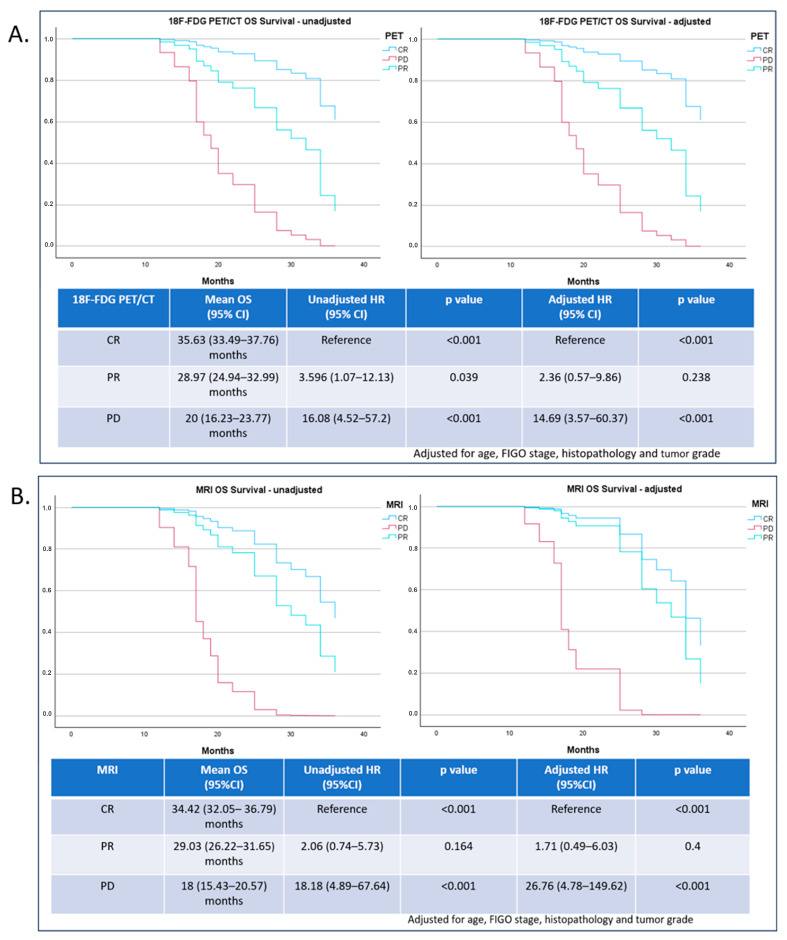
Overall survival as a function of 18F-FDG PET/CT (**A**) and MRI early therapy-response evaluation (**B**).

Mean PFS and OS times for 18F-FDG PET/CT and MRI are presented in Figure 3 and Figure 4, respectively. A statistically significant difference was noticed between PFS time for patients declared as PD (z value = 3.69; *p* < 0.0002).

As shown in Figure 3, the Cox regression PFS unadjusted hazard ratio (HR) for 18F-FDG PET/CT demonstrated a statistically significant connection between CR and both PR and PD (*p* < 0.034, and *p* < 0.014, respectively), differing from adjusted HR where a significant difference was observed between CR and PD, but not between CR and PR (*p* = 0.14). Lack of significance was established in both PFS unadjusted and adjusted HR for MRI between CR and PR (both *p* > 0.05).

A statistically significant impact was observed in all OS HR unadjusted ratios for 18F-FDG PET/CT. Adjusted HR significance was only observed between CR and PD, but not between CR and PR.

For MRI impact on overall survival (OS), a statistically significant difference was found between complete response (CR) and progressive disease (PD), for both unadjusted and adjusted hazard ratios (HR).

However, there was no significant difference found between CR and partial response (PR).

## 4. Discussion

Even though early post-treatment-response evaluation after CCRT is of great importance for further treatment management, exact and definite consensus about the time interval after the treatment and the choice between MRI and 18F-FDG PET/CT imaging modalities, and recently, also PET/MRI, has still not been achieved [24,25,26,27].

Therapy-response evaluation with PET/CT after CRT is not widely advocated nor accepted, possibly due to the shortage of definite evidence, but NCCN guidelines are still recommending 18F-FDG PET/CT as a preferred follow-up imaging modality, due to its ability to detect both locoregional and distant recurrence [9].

Apparently, a number of institutions adhere to recommendations, and conduct treatment response evaluations 3–6 months after the end of treatment, which is similar to our study concept, while some institutions perform the evaluation during the treatment [8,9].

The results of our study revealed good correspondence of 18F-FDG PET/CT results and fair correspondence of early MRI with follow-up examination results, with moderate agreement between MRI and 18F-FDG PET/CT data (Cohen’s kappa 0.6, 0.36, and 0.4, respectively), which is similar to the results of Peronne et al. who reported correspondence of PET/CT and follow-up of 0.84 and MRI and follow-up of 0.59 [28].

Lower sensitivity, specificity, NPV, and accuracy of early MRI examinations in comparison to 18F-FDG PET/CT results may be the consequence of a larger number of false-positive findings, in part possibly due to the difficulty of using early MRI to effectively differentiate post-treatment inflammatory changes from residual disease.

Since initial MRI examinations were performed earlier than 18F-FDG PET/CT (mean time 2.73 months for MRI; 3.63 months for PET/CT), it could be that the presence of posttreatment inflammatory changes caused by CCRT—oedema, necrosis, fibrosis, that may persist up to six months or even more—do result in a high risk of false-positive results, together with discrepancies in the detection of distant metastatic disease, and eventually, in a decrease in diagnostic-test characteristics.

We believe that the improvement in both 18F-FDG PET/CT and MRI diagnostic-test characteristics and the increase in specificity, PPV, and accuracy with time, that we observed, could be explained by the reduction in false positives and false negatives, and better detection of regional and distant metastatic involvement with progression of time [29,30,31].

Our results of MRI sensitivity and specificity for the detection of residual disease in comparison to 6-month control examinations (86.36% and 63.16%, respectively), resemble the study results of Vincens et al. who correlated the end-of-treatment MRI results with histopathological findings, with calculated sensitivity and specificity of 80% and 55%, respectively [32]. Results of the study conducted by Gui et al. reported low sensitivity and specificity of MRI with a high negative predictive value, which is in concordance with our results, suggesting that MRI itself may not be sufficient in fully accurately distinguishing posttherapy inflammation from residual cancer [31,33].

Though some studies reported low sensitivity for 18F-FDG PET/CT [34], our results indicate better sensitivity, specificity, NPV, and accuracy for 18F-FDG PET/CT (94.44%, 69.57%, 94.12%, and 80.49%, respectively), and better PPV of early MRI (73.08%), in concordance with the study of Su et al. which showed sensitivity, specificity, and accuracy of PET/CT (60%, 100%, and 89%, respectively), greater than that of MRI (27%, 100%, and 80%) [35]. The only statistical significance we determined was between the NPV of 18F-FDG PET/CT, which surpassed the NPV of MRI.

With the exception of lower MRI specificity, our results correlate to the study results of Dhesi et al. They observed better sensitivity of PET/CT and superior specificity of T2w and DWI with better PPV, and superior diagnostic accuracy and specificity for PET/MR compared with PET/CT, indicating the need for both PET/CT and MRI diagnostic-therapy-response evaluation information [35,36].

Though slightly higher, the relapse rate in CR patients determined by 18F-FDG PET/CT of 29.4% appears to be similar to the rate reported in other studies (ranging from 5 to 23%) [15,23,37].

Existing differences in relapse rate in CR patients are most probably the consequence of different study designs, and different posttreatment evaluation time points. Lack of accepted standardized qualitative and/or quantitative evaluation (like the “5-point scale”, proposed by Dhesi et al.) does not contribute to the uniformity of the reporting, but geographic distribution and health care system dissimilarities in LACC mortality rate must be taken into consideration [5,36,38].

Based on the calculated 76.5% 3-year OS, we are of the opinion that CR results determined by 18F-FDG PET/CT can serve as a good predictor of survival in treated patients with LACC, surpassing those of early MRI (60% 3-year OS).

Though we did not find this difference statistically significant, slightly better 18F-FDG PET/CT results in CR patients are rather coherent with the results of the study conducted by Beriwal et al. with a 3-year OS rate of 88%, suggesting that the introduction of PET/CT in routine posttherapy evaluation of LACC patients would be advisable [15,22,23,36,37].

Disease treatment management changes were enabled in 24.39% of patients due to distant metastatic presence detection by 18F-FDG PET/CT, which correlates with other available studies in the literature [39]. Furthermore, in 17.07% of the patients, the therapeutic approach was immediately altered, and further therapy was initiated after obtaining PD findings with both modalities. Re-irradiation was performed in 4.88% of patients, while the remaining 12.2% of patients underwent chemotherapy treatment.

At the end of the study, 56.11% of patients died, and out of the remaining patients at the 38-month checkpoint time, 36.58% were alive with no evidence of disease, and 7.31% with the disease. Results are worse than the European average of 62%, but comparable with expected relative survival in Eastern Europe with five-year relative survival of 57% [5,38].

Kaplan–Meier survival results revealed log-rank significant associations in both PFS and OS prediction for 18-F FDG PET/CT and early MRI results.

Our results for 18F-FDG PET/CT CR, PR, and PD showed PFS rates of 70.6%, 23.1%, and 0%, while for early MRI CR, PR, and PD, PFS rates were 53.3%, 36.8%, and 0%, respectively. OS rates for 18F-FDG PET/CT CR, PR, and PD were 76.5%, 43.9%, and 0%; and for MRI they were 60%, 47.4%, and 0%, respectively.

A number of prospective trials that evaluated FDG PET/CT in posttherapy response assessment found it to be predictive of patients’ survival [39,40,41].

18F-FDG PET/CT-based PFS and OS rate results in our study are comparable to Schwarz et al. study results, with a 3-year PFS rate for complete and partial metabolic response and progressive disease of 78%, 33%, and 0%, respectively [17].

Similar to other studies, we found that posttherapy evaluation demonstrating PD by both imaging methods does lead to high HR of cancer recurrence.

Meta-analysis by Kim et al. proved that 18F-FDG PET/CT could be a strong predictor of PFS and OS, declaring that the complete metabolic response had a significantly lower risk of progression and death compared with partial and progressive metabolic response [42].

Based on our results, early-response 18F-FDG PET/CT evaluation proved to be a potentially strong predictor of PFS and OS. Patients whose results were marked as PR or PD had a significantly higher risk of shorter PFS and OS time (PFS HR for PR was 3.94, and for PD, 10.32; for OS HR, it was 3.596, and for PD, 16.08). Early MRI evaluation showed that only the results marked as PD had a significant impact on risk for shorter PFS and OS time (HR for PFS was 10.6; and for OS, 18.18), indicating that the observed lack of significance for PR results was a probable repercussion of the false positives at an earlier MRI scanning checkpoint after CCRT, and that later MRI examination could be preferable in a follow-up of treated LACC patients. Also, we do recognize that the 18F-FDG PET/CT definition of treatment response is of higher importance in predicting outcomes.

The age of the patients has been revealed as a factor with significant negative influence on OS HR by adjusted Cox regression analysis, but only for early MRI results (*p* = 0.038), suggesting that an early MRI PD result in patients of younger age would have a significant predictive impact on shorter OS time.

After finding that the unadjusted HR for PR, as well as the adjusted HR for PR and PD based on 18F-FDG PET/CT, are more predictive for PFS and OS than early MRI results, we believe that the need for early implementation of 18F-FDG PET/CT in routine clinical practice is perceptible.

Even though some studies have not statistically confirmed the 18F-FDG PET/CT impact on PFS and OS, we agree with the statement that, at this point, no single modality can serve as a definitive predictive marker, and that multimodal imaging, combining 18F-FDG PET/CT and MRI techniques including DWI, or PET/MR will improve the follow-up of LACC patients [36].

The relatively small study size of only 41 patients was certainly a limiting factor in our study. A larger sample size would provide more reliable and generalizable results. We believe that the heterogeneity of the patient’s age and disease stage may also have influenced and/or biased the results of our study. A more homogeneous patient population or stratified analysis could additionally help in understanding the impact of examined variables on the outcomes. The technical lack of possibility to adjust the scanning schedule at exact time checkpoints in each patient also influenced the imaging assessment consistency, requiring cautiousness in result interpretation. On the other hand, the lack of accepted standardized evaluation criteria and standardized protocols led to additional variability in the interpretation of imaging results and affected the study’s reproducibility. We are also of the opinion that in order to capture long-term outcomes and late recurrences, longer follow-up may be necessary to fully ascertain the prognostic value of the PET/CT and MRI imaging modalities. Further research with the inclusion of a larger number of patients, a more homogeneous and stratified patient population, standardized evaluation criteria, and longer follow-up periods would be useful not only to validate and expand upon our results, but also in proposing the right time and/or modality for the control time checkpoints in order to maximally reduce the number of false-positive findings.

## 5. Conclusions

Our study demonstrates that 18F-FDG PET/CT does exceed MRI in the early assessment of therapeutic response in LACC patients after CCRT, serving as an equivalent diagnostic tool for local recurrence and distant metastatic disease detection.

Both 18F-FDG PET/CT and MRI provide information that may serve as predictive biomarkers of outcome and patients’ survival, both PFS and OS.

Implementation of 18F-FDG PET/CT as a diagnostic modality in everyday clinical practice, independently or in association with MRI can result in an improvement in therapeutic response assessment, providing the clinical conditions for a more accurate and personalized decision-making process in treated LACC patients, facilitating the acceleration of individualized disease treatment management.

Our study results endorse the stand that integration of MRI and 18F-FDG PET/CT modalities in the treatment response assessment of LACC patients is indispensable, considering the advantages and drawbacks of each separate imaging modality. Further prospective trials are needed to ensure the reliability of our findings and to provide additional evidence for optimal and standardized follow-up imaging, as future pillars of LACC patients care.

## Figures and Tables

**Table 1:** Patients and disease characteristics.

Female Patients (No. 41)		
Mean age 53.39 ± 11.85 years	Age range 31–77	Median 56
**FIGO Stage**	**No. of Patients (%)**	
IB2	2 (4.9%)	
IB3	1 (2.4%)	
IIA	4 (9.8%)	
IIA2	2 (4.9%)	
IIB	15 (36.6%)	
IIIC1	11 (26.8%)	
IIIC2	5 (12.2%)	
IVA	1 (2.4%)	
**Histopathological Subtype**	**No. of Patients (%)**	
Squamous carcinoma	36 (87.7%)	
Adenocarcinoma	4 (9.76%)	
Adenosquamous carcinoma	1 (2.44%)	
Duration between treatment response and 18F-FDG PET/CT	Mean Range	3.63 ± 0.54 2–4 months
Duration between treatment response and MRI	Mean Range	2.73 ± 0.45 2–3 months
Follow-up period	Median Range	27 months 12–38 months
**Disease Recurrence**	**No. of Patients (%)**	
Present	24 (58.5%)	
Absent	17 (41.5%)	
**Outcome**	**No. of Patients (%)**	
Deaths	23 (56.11%)	

**Table 2:** Diagnostic results at three observed checkpoints (early, at 6 months, and at 12-months control).

Diagnostic Results	Early MRI	18F-FDG PET/CT Early	MRI at 6 Months	MRI at 12 Months
CR ^1^	15 (36.6%)	17 (41.5%)	24 (58.5%)	17 (41.5%)
PR ^2^	19 (46.3%)	13 (31.7%)	4 (9.8%)	/
PD ^3^	7 (17.1%)	11 (26.8%)	13 (31.7%)	24 (58.5%)

^1^ CR—complete remission; ^2^ PR—partial remission; ^3^ PD—progressive disease.

**Table 3:** Crosstabulation of early MRI and early 18F-FDG PET/CT diagnostic results in comparison with the MRI results at 6 months and 12 months checkpoints.

Attribute	Early MRI vs. MRI at 6 Months (No.)	Early 18F-FDG PET/CT vs. MRI at 6 Months (No.)	Early MRI vs. MRI at 12 Months (No.)	Early 18F-FDG PET/CT vs. MRI at 12 Months (No.)
True positive	19	17	23	21
True negative	12	16	10	14
False positive	7	7	3	3
False negative	3	1	5	3
Sensitivity	86.36% 95% CI [65.09–97.09)	94.44% 95% CI [72.71–99.86]	82.14% 95% CI [63.11–93.94]	87.5% 95% CI [67.64–97.34]
Specificity	63.16% 95% CI [38.36–83.71]	69.57% 95% CI [47.08–86.79]	76.92% 95% CI [46.19–94.96]	82.35% 95% CI [56.57–96.2]
PPV ^1^	73.08% 95% CI [59.55–83.34]	70.86% 95% CI [56.45–81.98]	88.46% 95% CI [73.68–95.45]	87.5% 95% CI [71.26–95.18]
NPV ^2^	80% 95% CI [56.95–92.36]	94.12% 95% CI [70.03–99.1]	66.67% 95% CI [46.13–82.37]	82.35% 95% CI [61.28–93.22]
Accuracy	75.61% 95% CI [59.7–87.64]	80.49% 95% CI [65.13–91.18]	80.49% 95% CI [65.13–91.18]	85.37% 95% CI [70.83–94.43]

^1^ PPV—positive predictive value; ^2^ NPV—negative predictive value.

**Table 4:** Details of discordant cases.

No	18F-FDG PET/CT	Early MRI	6 Month Follow-Up MRI	Localisation/Status	12 Month Follow-Up MRI
1	PR	PR	CR	FU no evident disease	CR
2	PR	CR	CR	FU no evident disease	CR
3	PR	PR	CR	FU no evident disease	CR
4	CR	PR	CR	FU no evident disease	CR
5	PD	PR	CR	Early MRI—PR with parametrial infiltration 1 FU—CR 2 FU local relapse was proven	PD
6	PR	PR	CR	PET/CT and MRI residual cancer 1 FU—no evident disease 2 FU—progression with distant metastasis	PD
7	PR	PR	CR	1 FU—no evident disease 2 FU—progression with peritoneal involvement	PD
8	PR	PR	CR	Early partial response; PET/CT—suspected neck metastasis 2 FU—late local recurrence	PD
9	PR	CR	PR	1 FU—confirmed residual cancer; 2 FU—progression	PD
10	PR	CR	PD	PET/CT residual cancer 1 FU—progression	PD
11	CR	CR	PD	1 FU—residual cancer 2 FU—progression	PD
12	CR	CR	CR	2 FU—local recurrence	PD
13	CR	CR	CR	2 FU—local recurrence	PD

Abbreviations: FU—follow-up; CR—complete response; PR—partial response; PD—progressive disease.

**Table 5:** 18F-FDG PET/CT, early, six-, and twelve-months control MRI PFS and OS rates.

PFS Rate	CR	PR	PD
18F-FDG PET/CT	70.6%	23.1%	0%
Early MRI	53.3%	36.8%	0%
6-month MRI	62.%	0%	0%
12-month MRI	88.2%	/	0%
**OS Rate**	**CR**	**PR**	**PD**
18F-FDG PET/CT	76.5%	38.5%	0%
Early MRI	60%	47.4%	0%
6-month MRI	70.8%	25%	0%
12-month MRI	88.2%	/	12.5%

## Data Availability

The data that support the finding of this study are available from the corresponding author (SL) upon reasonable request.
